# Development of a Zimbabwean child growth curve and its comparison with the World Health Organization child growth standards

**DOI:** 10.4102/phcfm.v14i1.3278

**Published:** 2022-09-13

**Authors:** Anesu Marume, Archary Moherndran, Partson Tinarwo, Saajida Mahomed

**Affiliations:** 1 School of Nursing and Public Health, College of Health Sciences, University of KwaZulu-Natal, Durban, South Africa; 2 Ministry of Health and Child Care Zimbabwe, Health Promotion, Government of Zimbabwe, Harare, Zimbabwe; 3 Department of Padeatrics, College of Health Sciences, University of KwaZulu-Natal, Durban, South Africa; 4 King Edward VIII Hospital, Durban, South Africa; 5 School of Laboratory Medicine and Medical Sciences, College of Health Sciences, University of KwaZulu-Natal, Durban, South Africa

**Keywords:** children, LMS method, growth curve, obesity, smooth growth curves, stunting, WHO growth standards, Zimbabwe

## Abstract

**Background:**

There is limited research that describes the growth trajectories of African children. The development of World Health Organization (WHO) growth standards considered a sample of children who lived in environments optimum for human growth.

**Aim:**

This study aimed to develop weight-for-age and height-for-age growth curves from the Zimbabwean 2018 National Nutrition Survey and compare them with the WHO growth standards.

**Setting:**

Study participants were recruited from all districts in Zimbabwe.

**Methods:**

Height-for-age and weight-for-age data collected from 32 248 children were used to develop the Zimbabwean references. Smooth growth curves (height, weight and body mass index [BMI]-for-age) were estimated with the Lambda Mu Sigma (LMS) method and compared with the WHO growth standards.

**Results:**

Zimbabwean children were shorter and weighed less in comparison with the WHO growth standards. The –2 standard deviation (s.d.) Z-score curves (height-for-age) for Zimbabwean children (boys and girls) were below the –1 s.d. Z-score curves of the WHO growth standards. The Zimbabwean Z-scores (BMI-for-age) values above –1 s.d. were significantly higher in comparison with the corresponding WHO growth standards.

**Conclusion:**

Utilising the WHO growth standards would diagnose a higher proportion of Zimbabwean children as stunted whilst underestimating the proportion at risk of obesity. The WHO growth standards lack a consideration of the geographical, economic, political and environmental constraints existing between countries.

## Introduction

Growth curves are useful tools in assessing the health, nutrition and overall growth of a child. Periodic measurements of children’s heights and weights and comparison with age-specific expected heights and weights provide health workers and caregivers with an opportunity to identify any nutrition or growth complications. The World Health Organization (WHO) growth standards are the most widely used growth curves globally.^1^ These growth standards refer to a set of curves designed to estimate the normal child growth trajectory with cut-off points to identify deviation from the normal.

The WHO growth standards were developed from the analysis of a sample of children in six countries. Children enrolled in the study were selected from environments that were considered optimal for ideal child growth.^2^ Children were enrolled from communities with low infant mortality rates, a high water and sanitation coverage, low altitude (< 1500 m) with low population mobility. Furthermore, mothers of the enrolled children were expected to follow international feeding recommendations.^3^ In developing the growth standards a cohort of healthy, first-time pregnancy and non-smoking mothers were followed up from pregnancy up to 24 months of the child’s age.^3^ Only data from children who had grown within the conditions set in the inclusion criteria were considered for analysis in both the longitudinal and cross-sectional studies conducted. Data from children who experienced morbidities with a potential to affect child growth such as diarrhoea, malaria, haemolytic anaemia, glucose-6-phosphate dehydrogenase (G6PD) deficiency, Crohn’s disease, renal tubule-interstitial disease and protein-energy malnutrition were not analysed.^4,5^

The strict inclusion criteria used when developing the WHO growth standards has resulted in the tool having a very high sensitivity and a very low specificity for malnutrition.^6,7^ The optimal conditions required in the cohorts during development of the WHO growth standards may be aspirational; however, they do not reflect the situation in communities in a large proportion of the world. It is therefore difficult to generalise the WHO growth standards to all populations, especially those living in countries with high infant mortality and poor water and sanitation coverage. These environmental differences have a significant impact on child growth. People living in impoverished environments were observed to significantly present with a small intestine abnormality, a condition termed environmental enteropathy or enteric dysfunction.^8,9^ Multiple studies have found a strong link between environmental enteropathy and stunting.^10,11,12^ Using WHO growth standards in assessing children’s anthropometric measurements without addressing the inequity highlighted will most likely result in underestimation or overestimation of malnutrition.

There have been multiple studies that have validated the WHO child growth standards to assess their appropriateness within specific settings and contexts. The height-for-age Z-scores (HAZ) have the most notable difference by settings. The HAZ distribution in low- and middle-income countries (LMICs) has a downward shift for the entire distribution, indicative of a slower growth for children in these countries.^13^ Amongst the literature analysed in a review of publications that validated WHO child growth standards globally, none presented similar growth trajectories as those estimated by WHO child growth standards.^14^

There have been multiple studies that have assessed the differences between the WHO growth standards and local populations in Europe, America and Asia. However, there is a paucity of similar studies in the WHO-Africa region. Amongst the studies identified in a literature review of global validations of the WHO growth standards only one African study focused on children within the ages 0–5 years.^14^ The WHO-Africa region has the highest prevalence of children with stunting and is amongst the most affected regions for nearly all forms of malnutrition. The aim of this study was to develop weight-for-age and height-for-age growth curves from the Zimbabwean 2018 National Nutrition Survey and compare these curves with the WHO growth standards to assess the applicability of the WHO child growth standards in evaluating nutrition outcomes of children in Zimbabwe. This study is therefore expected to add an African perspective to the growing body of literature on child growth standards and references.

## Subjects and methods

### Study design

A cross-sectional survey (The National Nutrition Survey) was conducted in January and February 2018 in Zimbabwe to assess the nutritional status of children based on their weight and height.^15^ The Ministry of Health and Child Care, Zimbabwe granted permission to access raw data from the 2018 National Nutrition Survey. Ethical approval was granted by the University of KwaZulu-Natal (UKZN) Biomedical Research Ethics Committee (BREC) [BE109/19] and the Medical Research Council of Zimbabwe (MRCZ) [MRCZ/A/2479].

### Setting

Zimbabwe is divided into 10 administrative provinces, which are further subdivided into 59 districts. The National Nutrition Survey considered children from all the 59 districts in Zimbabwe.^15^

### Population and sampling strategy

Each district in Zimbabwe was divided into 30 enumeration areas (EAs) based on the 2012 census with the use of the probability proportional to population size (PPS) method. Villages within the EA were systematically selected to ensure equal representation. Thirty households were to be enumerated from each EA. Households with children below the age of five years were considered the sampling unit. A household with no child under five years was replaced with the nearest household. All children under five years living in the household had their anthropometric measurements taken. A total of 28 464 households were reached and interviewed. Anthropometric measurements of 34 714 children aged between 6 and 59 months were carried out.^15^

### Procedure

Data collectors and enumerators were trained to ensure the precision and accuracy of anthropometric measurements. Measuring instruments (scales and height boards) were calibrated daily with regular supervision from the provincial and district nutritionists. The weight-for-age and height-for-age data measured during the National Nutrition Survey were used in this study to generate the child growth curves for the Zimbabwean population. Records without complete information for weight, height and age were not included. Children whose weight-for-age and HAZ scores > 5 were considered as outliers according to the WHO growth standards guidelines.^4^ The final sample analysed comprised 31 369 children.

### Data analysis

The generalized additive models for location scale and shape (GALMSS) method was used to develop growth curves. The lambda mu sigma (LMS) method that estimates the gender-specific percentile curves was used to smoothen the growth curves. This method is premised on an assumption that anthropometric data can be converted to a standard normal distribution through the use of a Box-Cox transformation for any given age.^16^ It is based on three curves representing the skewness (L), the median (M) and the coefficient of variation (S) of the original data as they vary with age. The parameters L, M and S are fitted as a function of age by cubic splines.^17^ The LMS method allows for anthropometric indicators (height or weight) for each child’s age and gender to be converted directly to standard deviation scores (s.d.-scores, which is synonymous to Z-scores) that follow a standard normal distribution. The following formula was used to calculate the Z-score measure, y (either weight, height or body mass index [BMI]) at time *t* from the smooth curve L(*t*), M(*t*) and S(*t*):


$$Z=\frac{{\left[\frac{y}{M(t)}\right]}^{L(t)}-1}{S(t)L(t)}$$ (Eqn 1)

The ±1, ±2, ±3 and median Z-scores for Zimbabwean children were compared with the WHO growth standards for height-for-age, weight-for-age and weight-for-height. Data that had Z-scores >3 were dropped in line with the Ministry of Health and Child Care, Zimbabwe guidelines.^18^ As a result of the skewness of the data, a Wilcoxon test was used to assess the significance of the median difference between the Zimbabwean growth curves and the WHO growth standards. Both the descriptive and inferential statistics were conducted in R Statistical Computing software, 3.6.3 of the R Core Team, 2020 using the R Studio environment. All the tests were conducted at the 5% level of significance.

### Ethical considerations

The Ministry of Health and Child Care, Zimbabwe granted permission to access raw data from the 2018 National Nutrition Survey. Ethical approval was granted by the University of KwaZulu-Natal (UKZN) Biomedical Research Ethics Committee (BREC) [BE109/19] and the Medical Research Council of Zimbabwe (MRCZ) [MRCZ/A/2479].

## Results

A majority of the population were girls (51%). The median age of the children was 32.35 months (interquartile range [IQR]: 19.52–45.11) months. Boys had a median age of 32.46 months (IQR: 19.48–45.11) and girls’ median age was 32.23 months (IQR: 19.52–45.14); (*p* = 0.445).

The LMS values, length-for-age, weight-for-age and BMI-for-age Z-scores for Zimbabwe children as calculated from the National Nutrition Survey data set are presented in online Appendix 1 Tables 1-A1, 2-A1 and 3-A1. Zimbabwean children were shorter and weighed less in comparison with the corresponding WHO growth standards (Table 1).^19^ Zimbabwean boys were significantly more likely to have different Z-score values in comparison with the corresponding WHO growth standards Z-score values than the Zimbabwean girls.

**TABLE 1:** Mean difference between Zimbabwean children and World Health Organization growth standards.

Z-score	Boys				*p*	Girls				*p*
	WHO		Zimbabwe			WHO		Zimbabwe		
	Median	Q1–Q3	Median	Q1–Q3		Median	Q1–Q3	Median	Q1–Q3	
Height-for-age										
–3 s.d.	83.1	75.2–89.7	77.3	70.5–83.2	0.003	81.6	73.0–88.8	73.0	66.3–82.3	0.002
–2 s.d.	86.7	77.9–93.9	78.3	71.0–87.0	< 0.001	85.3	76.0–93.0	79.6	71.5–86.9	0.007
–1 s.d.	90.2	80.7–98.0	83.9	75.5–92.3	0.008	89.0	79.0–97.2	84.6	74.9–91.1	0.036
0	93.8	83.5–102	88.5	80.1–97.2	0.045	92.6	82.0–101	88.7	79.2–97.4	0.144
1 s.d.	97.3	86.3–106	92.3	83.3–102	0.072	96.2	85.0–106	91.3	83.0–101	0.142
2 s.d.	101	89.1–110	96.2	85.8–106	0.138	99.9	87.9–110	96.7	87.3–105	0.233
3 s.d.	104	91.8–114	98.1	91.3–110	0.222	104	90.9–114	99.9	91.1–109	0.502
Weight-for-age										
–3 s.d.	9.65	8.03–11.0	8.85	7.70–10.2	0.062	9.20	7.35–10.7	8.60	6.93–10.0	0.096
–2 s.d.	10.9	8.95–12.5	9.60	7.63–11.6	0.013	10.4	8.25–12.1	9.65	7.58–11.6	0.174
–1 s.d.	12.2	10.0–14.1	10.9	8.85–12.8	0.012	11.7	9.25–13.7	10.9	8.93–12.7	0.142
0	13.8	11.2–16.0	12.5	9.75–14.2	0.023	13.2	10.5–15.7	12.4	10.2–14.4	0.110
1 s.d.	15.5	12.6–18.2	13.8	11.6–16.3	0.013	15.0	11.9–18.0	13.8	11.1–16.0	0.037
2 s.d.	17.5	14.0–20.7	15.6	12.6–17.5	0.004	17.2	13.6–20.9	15.6	12.6–18.0	0.028
3 s.d.	19.8	15.7–23.5	16.0	13.8–19.2	0.001	19.8	15.5–24.4	17.1	14.0–19.8	0.009
BMI-for-age										
–3 s.d.	12.5	12.1–12.9	11.8	11.5–12.2	< 0.001	12.2	11.9–12.3	12.0	11.8–12.3	0.085
–2 s.d.	13.5	13.1–13.8	13.3	13.0–13.6	0.022	13.1	12.9–13.3	13.3	12.9–13.7	0.203
–1 s.d.	14.6	14.2–14.9	14.6	14.1–15.0	0.425	14.2	14.0–14.4	14.5	14.1–14.7	0.018
0	15.7	15.4–16.1	15.8	15.5–16.5	0.286	15.4	15.3–15.7	15.7	15.3–16.3	0.145
1 s.d.	17.0	16.7–17.4	17.3	16.9–18.1	0.036	16.9	16.8–17.1	17.4	16.8–18.0	0.020
2 s.d.	18.5	18.2–18.9	19.2	18.4–19.7	0.014	18.6	18.5–18.8	19.2	18.5–20.0	0.012
3 s.d.	20.2	19.9–20.7	21.5	20.4–22.2	< 0.001	20.6	20.4–21.0	21.3	20.6–22.2	0.002

*Source:* Adapted from World Health Organization. The WHO Child Growth Standards [homepage on the Internet]. No date. Available from: https://www.who.int/tools/child-growth-standards/standards

BMI, body mass index; s.d., standard deviation; WHO, World Health Organization.

### Height-for-age

**FIGURE 1:**
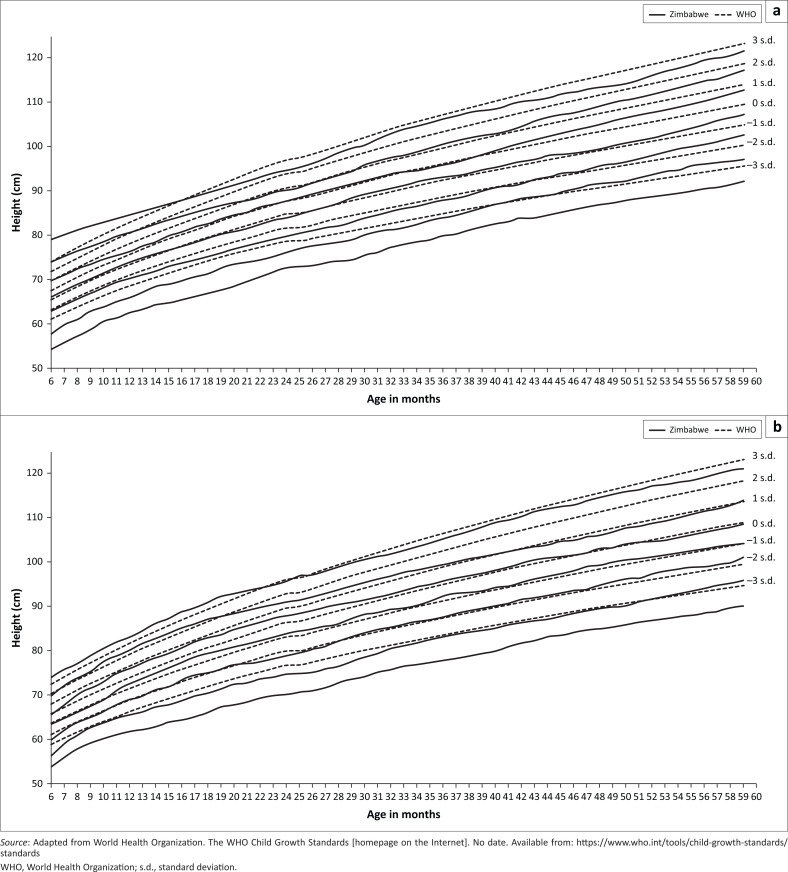
Height-for-age graphs for children 6–59 months, Zimbabwe.

Both the Zimbabwean boys and girls had height-for-age growth below the WHO growth standards (Figure 1). The lower curves (0 s.d., −1 s.d., 2 s.d. and 3 s.d. Z-score lines) for Zimbabwe were 1 s.d. below the WHO growth standards for both boys and girls. The 2 s.d. and 3 s.d. growth curves for Zimbabwean boys aged 6–12 months were higher than the corresponding WHO growth standards growth curves. The 2 s.d. and 3 s.d. growth curves for Zimbabwean girls intercepted the WHO growth standards at the age of 18 months and 22 months, respectively. The median height for the –2 s.d. Z-score for Zimbabwean children was significantly lower than the corresponding WHO growth standards for both boys (*p* < 0.001) and girls (*p* = 0.007) (Table 1). The –2 s.d. Z-score curve used to identify stunting in children was also below the –1 s.d. Z-score curve for the WHO growth standards. Using the WHO growth standards is significantly more likely to identify a higher proportion of children as stunted (*p* < 0.001).

### Weight-for-age

**FIGURE 2:**
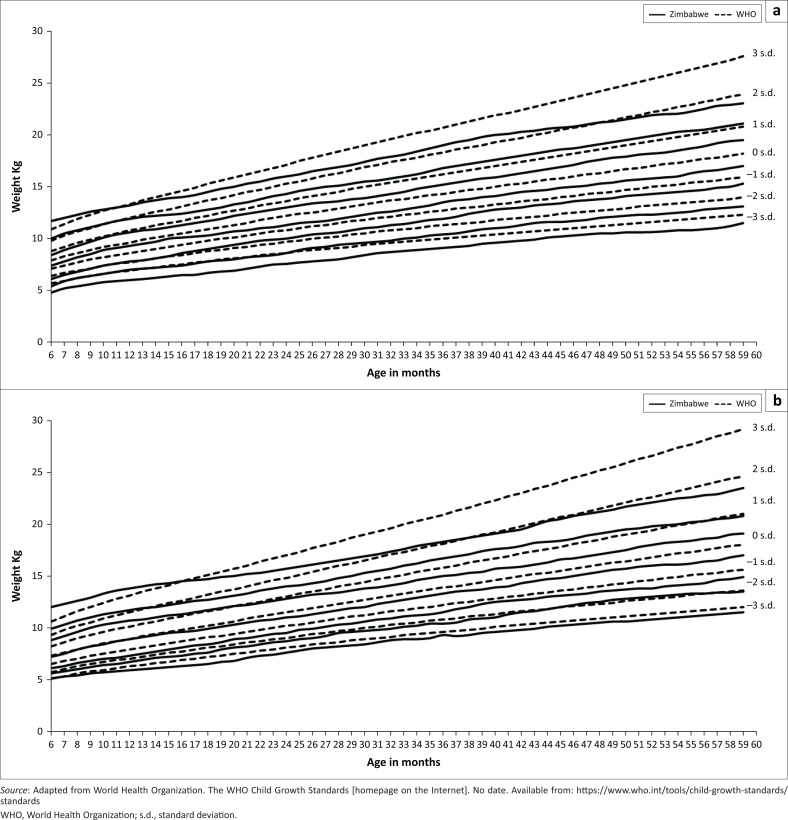
Weight-for-age graphs for children 6–59 months, Zimbabwe.

Zimbabwean girls’ growth curves had a pattern that was significantly similar to the WHO growth standards for all Z-scores below the median (Table 1). In contrast, Zimbabwean boys had significantly lower weight-for-age Z-score values in comparison with the corresponding WHO growth standards. For the Z-scores 1 s.d., 2 s.d. and 3 s.d., Zimbabwean girls had weight-for-age outcomes below the WHO growth standards. The 3 s.d. Z-score curve for both boys and girls intercepted the WHO growth standards 2 s.d. Z-score curve at 44, 39 months, respectively, as shown in Figure 2. For Zimbabwean boys, the –2 s.d. Z-score curve was below the –3 s.d. Z-score curve from ages 6–24 months with exhibited growth catch-up that still remained below the corresponding –2 s.d. Z-score curve on the WHO growth standards.

### Body mass index for age

There was no significant difference between the Zimbabwean and WHO growth standards median Z-score curve for both boys and girls. Zimbabwean girls had higher BMI values in comparison with the corresponding WHO growth standards. From the median Z-score curve to –3 s.d. Z-score, the growth of Zimbabwean girls estimated the WHO growth standards. For Zimbabwean boys, the lower Z-score curves (–2 s.d. and –3 s.d.) had significantly lower BMI outcomes in comparison with WHO growth standards whilst the Z-score curves above the median were significantly above the corresponding WHO growth standards. For both genders, the Zimbabwean Z-scores values above –1 s.d. were significantly higher in comparison with the corresponding WHO growth standards.

## Discussion

This is the first study to compare the growth of Zimbabwean children with the WHO growth standards. The WHO growth standards had higher height-for-age and weight-for-age outcomes in comparison with the Zimbabwean-generated growth curves. The WHO growth standards BMI-for-age Z curves were lower in comparison with the corresponding growth curves for the Zimbabwean sample.

The –2 s.d. Z-score curve for Zimbabwe is below the –3 s.d. Z-score curve of the WHO growth standards, which would result in children identified as moderately stunted using the Zimbabwean growth curve being classified as severely stunted using the WHO growth standards. Zimbabwe is classified as a low-income country.^20^ Low-income countries are characterised with poor dietary diversity and environmental constraints that inhibit child growth.^21^ The WHO growth standards were designed to represent optimum child growth patterns, and thus it is more likely to classify children living in countries such as Zimbabwe as stunted when they are of normal height.^22,23^

The weight-for-age curves for Zimbabwean children were lower than their corresponding curves in the WHO growth standards. In a comparison of WHO and Centre for Disease Control (CDC) growth curves, differences in weight-for-age were argued to be as a result of different feeding patterns between children in the populations used to develop these curves.^2^ Body mass index is mostly used to identify obesity in adults. In children, it is used as a screening tool for possible obesity and hence as a measure of adiposity. Zimbabwean children had higher BMI scores in comparison with the WHO child growth standards. These higher scores may suggest that Zimbabwean children are at a high risk of childhood obesity.

A comparison of Polish children’s growth with WHO growth standards found similar results, which suggest that the BMI-for-age distribution has had an upward shift because of changes in diet and culture.^24^ Another study conducted in Poland argued that the difference observed in the BMI-for-age distribution may be attributed to a difference in the proportion of children reported to have gone through exclusive breastfeeding.^25^ The Zimbabwe Demographics of Health Survey (ZDHS) estimates that less than 50% of Zimbabwean children are exclusively breastfed.^26^

We found that Zimbabwean girls were more likely to grow within the WHO growth standards expected trajectory compared with boys. Studies conducted in Malaysia, South Africa and Poland have reported similar results.^21,24,27^ A study conducted in China that found the growth of Chinese girls to be similar to the WHO growth standards in contrast with the growth of Chinese boys, suggested that families were more likely to monitor and moderate weight for girls in order to preserve the lean body in comparison with boys who culturally expect to be strong.^28^ A study in the Democratic Republic of Congo in which girls were more likely to have a growth curve that imitates WHO growth standards argued that African boys were exposed to labour-intensive activities, which may not be matched with additional dietary intake.^29^ The same study further argued that boys were more likely to be exposed to environmental stressors because of their outdoor activities, which strain their immunity.

Amongst the limited studies in Africa that validated WHO growth standards against local growth patterns, there is a general agreement that children in Africa have growth patterns lower than the WHO child growth standards.^27,29,30^ Regional agreements are also observed with Asian countries (except China) reporting shorter children, European countries having taller and heavier children and North and South American countries (except the United States and Canada) reporting shorter children in comparison with WHO growth standards.^15^ A study in Argentina argues that regional differences are mostly explained by epigenetic interactions between genes and environmental factors.^31^ Environmental and geographical factors are often present in numerous countries within a region.

From the graphs presented, it is apparent that the WHO growth standards can overdiagnose underweight and stunting amongst Zimbabwean children. Evidence has shown that Zimbabwe has environmental, economic and genetic factors, which may predispose children to the low anthropometric outcomes observed.^22,23^ Relying on the WHO growth standards that imply ideal child growth may result in some children being referred for management of malnutrition when they have attained their highest possible outcome within the existing conditions. An evaluation of the child growth monitoring programme in Zimbabwe reported that the system is overburdened, thus failing to cater for all the children referred for management of malnutrition.^32^ A growth curve that removes environmental and genetic factors, which are beyond the health system can reduce the unnecessary burden on the health system in Zimbabwe. Future studies may do well to assess the implications of the possible misclassification of children in terms of the healthcare of these children and caregiving by their guardians.

**FIGURE 3:**
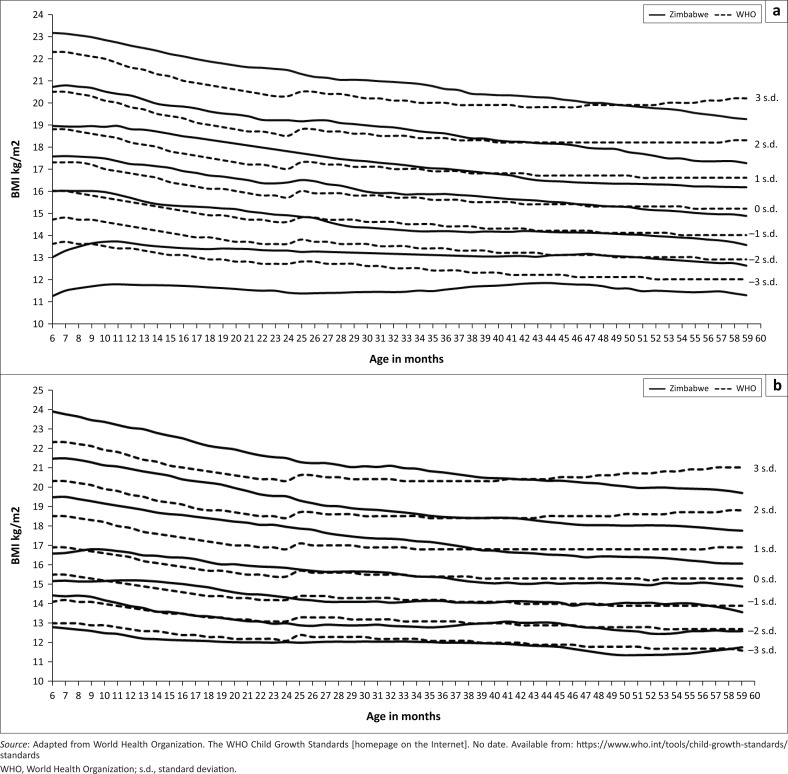
Body mass index-for-age graphs for children 6–59 months, Zimbabwe.

The WHO child growth standards represent ideal child growth and it is ideal for all children globally, for growth under optimum conditions. This is in line with the Sustainable Development Goals and the Nutrition Global targets. However, global conditions under which children grow vary immensely with inequalities in income, access to diet and cultural diversity. Thus, the development of regional growth references is more likely to reflect the environmental, geographical and sometimes socio-economic influences on child growth. Furthermore, regional growth reference may be more appropriate in order to identify interventions to reduce adverse nutrition outcomes amongst children.

### Strengths and limitations

The data set used in this study is nationally representative of Zimbabwean children and did not exclude children based on socio-economic or feeding practices. The growth curves developed based on local data can be used to assess changes in population anthropometrics over time. The national data set may have included children with birth defects that can result in diminished or excess growth. The growth curve developed was based on data collected as part of a cross-sectional survey, whereas the WHO growth standards contained a longitudinal component. Therefore, the differences between the curves must be interpreted with regard to the possible limitations. A study that utilises the inclusion criteria considered in the development of the WHO growth standards can be used to confirm our findings.

## Conclusion

The growth curves presented in this study describe and compare the growth pattern of Zimbabwean Children to the WHO child growth standards. Zimbabwean children have height-for-age and weight-for-age growth that is significantly below the WHO child growth standards. The WHO child growth standards are more likely to identify a higher proportion of children with undernutrition and miss a larger proportion of children with overnutrition. Growth monitoring provides an essential tool to meet the sustainable development goal of eliminating forms of childhood malnutrition. Whilst the WHO growth standards ensure timely identification of undernutrition in Zimbabwe, a growth reference that takes into consideration differences by acknowledging environmental constraints is more likely to provide an accurate diagnosis and advise effective interventions. Using WHO growth standards in this region may result in classifying a healthy child as stunted when they have attained their highest possible height, taking into consideration both their genetic and environmental constraints present.

## References

[1] De Onis M, Onyango A, Borghi E, et al. Worldwide implementation of the WHO child growth standards. Public Health Nutr. 2012;15(9):1603–1610. 10.1017/S136898001200105X 22717390

[2] De Onis M, Garza C, Onyango AW, Borghi E. Comparison of the WHO child growth standards and the CDC 2000 growth charts. J Nutr. 2007;137(1):144–148. 10.1093/jn/137.1.144 17182816

[3] De Onis M, Onyango AW, Borghi E, Garza C, Yang H, Group WMGRS. Comparison of the World Health Organization (WHO) child growth standards and the National Center for Health Statistics/WHO international growth reference: Implications for child health programmes. Public Health Nutr. 2006;9(7):942–947. 10.1017/PHN20062005 17010261

[4] WHO MGRS WMGRS, De Onis M. WHO child growth standards based on length/height, weight and age. Acta Paediatr. 2006;95:76–85. 10.1111/j.1651-2227.2006.tb02378.x 16817681

[5] MGRS, De Onis M. Assessment of differences in linear growth among populations in the WHO multicentre growth reference study. Acta Paediatr. 2006;95:56–65. 10.1111/j.1651-2227.2006.tb02376.x 16817679

[6] Ahmad UN, Yiwombe M, Chisepo P, Mwalwanda S, Heikens T, Kerac M. A randomised controlled trial exploring how new who growth charts influence healthcare workers’ clinical decisions and recommendations about exclusive breastfeeding for infants aged < 6 months. Arch Dis Child. 2011;96(Suppl 1):A73–A73. 10.1136/adc.2011.212563.170

[7] Khadilkar V. The growing controversy about growth charts: WHO or regional? Int J Pediatr Endocrinol. 2013;2013(1):O6. 10.1186/1687-9856-2013-S1-O6

[8] Kosek MN, Ahmed T, Bhutta Z, et al. Causal pathways from enteropathogens to environmental enteropathy: Findings from the MAL-ED birth cohort study. EBioMedicine. 2017;18:109–117. 10.1016/j.ebiom.2017.02.024 28396264 PMC5405169

[9] Watanabe K, Petri Jr WA. Environmental enteropathy: Elusive but significant subclinical abnormalities in developing countries. EBioMedicine. 2016;10:25–32. 10.1016/j.ebiom.2016.07.030 27495791 PMC5006727

[10] Guerrant RL, Leite AM, Pinkerton R, et al. Biomarkers of environmental enteropathy, inflammation, stunting, and impaired growth in children in northeast Brazil. PLoS One. 2016;11(9):e0158772. 10.1371/journal.pone.0158772 27690129 PMC5045163

[11] Harper KM, Mutasa M, Prendergast AJ, Humphrey J, Manges AR. Environmental enteric dysfunction pathways and child stunting: A systematic review. PLoS Negl Trop Dis. 2018;12(1):e0006205. 10.1371/journal.pntd.0006205 29351288 PMC5792022

[12] Vonaesch P, Randremanana R, Gody J-C, et al. Identifying the etiology and pathophysiology underlying stunting and environmental enteropathy: Study protocol of the AFRIBIOTA project. BMC Pediatr. 2018;18(1):236. 10.1186/s12887-018-1189-5 30025542 PMC6053792

[13] Roth DE, Krishna A, Leung M, Shi J, Bassani DG, Barros AJ. Early childhood linear growth faltering in low-income and middle-income countries as a whole-population condition: Analysis of 179 demographic and health surveys from 64 countries (1993–2015). Lancet Glob Health. 2017;5(12):e1249–e1257. 10.1016/S2214-109X(17)30418-7 29132614 PMC5695758

[14] Marume A, Archary M, Mahomed S. Validation of growth standards and growth references: A review of literature. J Child Health Care. 2021. 10.1177/13674935211024816 34114485

[15] Food and Nutrition Council. Zimbabwe National Nutrition Survey 2018 Report [homepage on the Internet]. 2018 [cited 2018 Dec 12]. Available from: https://www.unicef.org/zimbabwe/media/1056/file/Zimbabwe%202018%20National%20Nutrition%20Survey%20Report.pdf

[16] Cole TJ. The development of growth references and growth charts. Ann Hum Biol. 2012;39(5):382–394. 10.3109/03014460.2012.694475 22780429 PMC3920659

[17] Flegal KM, Cole TJ. Construction of LMS parameters for the Centers for Disease Control and Prevention 2000 growth charts. United States: Department of Health and Human Services; Centers for Disease Control and Prevention; National Center for Health Statistics; 2013. 24992748

[18] MOHCC. Guidelines for management of acute malnutrition through community-based therapeutic care (CTC) [homepage on the Internet]. 2008 [cited 2021 Dec 2]. Available from: https://www.humanitarianresponse.info/sites/www.humanitarianresponse.info/files/documents/files/Guidelines%20for%20Management%20of%20Acute%20Malnutrition%20through%20Community-based%20Therapeutic%20Care%20%28CTC%29.pdf

[19] World Health Organization. The WHO Child Growth Standards [homepage on the Internet]. No date. Available from: https://www.who.int/tools/child-growth-standards/standards

[20] World Bank. Overcoming economic challenges, natural disasters, and the pandemic: Social and economic impacts [homepage on the Internet]. 2021 [cited 2021 Dec 02]. Available from: https://documents1.worldbank.org/curated/en/563161623257944434/pdf/Overcoming-Economic-Challenges-Natural-Disasters-and-the-Pandemic-Social-and-Economic-Impacts.pdf

[21] Bong Y, Shariff AA, Mohamed AM, Merican AF. Malaysian growth centiles for children under six years old. Ann Hum Biol. 2015;42(2):109–116. 10.3109/03014460.2014.912679 24853607

[22] Cameron N, Hawley NL. Should the UK use WHO growth charts? Paediatr Child Health. 2010;20(4):151–156. 10.1016/j.paed.2009.11.001

[23] Piaggio U. Question 1: UK-WHO versus customised growth charts for the identification of at-risk small for gestational age infants: Which one should we use? Arch Dis Child. 2018;103(4):399–401. 10.1136/archdischild-2017-313679 29273646

[24] Kulaga Z, Litwin M, Tkaczyk M, et al. Polish 2010 growth references for school-aged children and adolescents. Eur J Pediatr. 2011;170(5):599–609. 10.1007/s00431-010-1329-x 20972688 PMC3078309

[25] Kulaga Z, Grajda A, Gurzkowska B, et al. Polish 2012 growth references for preschool children. Eur J Pediatr. 2013;172(6):753–761. 10.1007/s00431-013-1954-2 23371392 PMC3663205

[26] ZIMSTAT, ICF International. Zimbabwe demographic and health survey 2015: Final report. Rockville, MD: ZIMSTAT, ICF International; 2016.

[27] Nyati LH, Pettifor JM, Norris SA. The prevalence of malnutrition and growth percentiles for urban South African children. BMC Public Health. 2019;19(1):1–13. 10.1186/s12889-019-6794-1 31046727 PMC6498578

[28] Zong X-N, Li H. Construction of a new growth references for China based on urban Chinese children: Comparison with the WHO growth standards. PLoS One. 2013;8(3):e59569. 10.1371/journal.pone.0059569 23527219 PMC3602372

[29] Buhendwa RA, Roelants M, Thomis M, Nkiama CE. Nutritional status and height, weight and BMI centiles of school-aged children and adolescents of 6–18-years from kinshasa (DRC). Ann Hum Biol. 2017;44(6):554–561. 10.1080/03014460.2017.1333149 28535703

[30] Garzón M, Papoila AL, Alves M, Pereira-da-Silva L. Comparison of growth curve estimates of infants in Sao Tome Island, Africa, with the WHO growth standards: A birth cohort study. Int J Environ Res Public Health. 2019;16(10):1693. 10.3390/ijerph16101693 PMC6572562 31091793

[31] Orden AB, Apezteguía MC. Weight and height centiles of Argentinian children and adolescents: A comparison with WHO and national growth references. Ann Hum Biol. 2016;43(1):9–17. 10.3109/03014460.2014.970576 25350773

[32] Marume A, Mafaune P, Maradzika J, January J. Evaluation of the child--growth-monitoring programme in a rural district in Zimbabwe. Early Child Dev Care. 2017;189(2):318–327. 10.1080/03004430.2017.1320784

